# A Contemporary Multidimensional Insight into the Clinical and Pathological Presentation of Urological Conditions Associated with HIV: A Narrative Review

**DOI:** 10.3390/tropicalmed10110318

**Published:** 2025-11-11

**Authors:** Hannah Faherty, Jamshaid Nasir Shahid, Yousef Abu Osba, Maryam Jamshaid, Dushyant Mital, Mohamed H. Ahmed

**Affiliations:** 1 Department of Surgery, Milton Keynes University Hospital NHS Foundation Trust, Milton Keynes MK6 5LD, UK; hannah.faherty@mkuh.nhs.uk; 2 Department of Urology, Bedford Hospital NHS Foundation Trust, Bedford MK42 9DJ, UK; jamshaid.shahid@bedsft.nhs.uk; 3 Department of Surgery, Leicester University Hospital, Leicester LE1 5WW, UK; yousef.abuosba@uhl-tr.nhs.uk; 4 Department of Surgery, Liverpool University Hospital, Liverpool L7 8YE, UK; maryamjamshaid9819@gmail.com; 5 Department of Blood Borne Virus and HIV, Milton Keynes University Hospital NHS Foundation Trust, Eaglestone, Milton Keynes MK6 5LD, UK; dushyant.mital@mkuh.nhs.uk; 6 Department of Geriatric Medicine, Milton Keynes University Hospital NHS Foundation Trust, Eaglestone, Milton Keynes MK6 5LD, UK; 7 Faculty of Medicine and Health Sciences, University of Buckingham, Buckingham MK18 1EG, UK; 8 Department of Medicine and HIV Metabolic Clinic, Milton Keynes University Hospital NHS Foundation Trust, Eaglestone, Milton Keynes MK6 5LD, UK

**Keywords:** HIV, urology, urinary tract infection, malignancy, sexually transmitted disease

## Abstract

Human Immunodeficiency Virus (HIV) infection is associated with a wide spectrum of urological manifestations, reflecting both the direct effects of viral infection and the indirect consequences of immunosuppression, opportunistic infections, malignancies and long-term combined antiretroviral therapy (cART). This narrative review provides a contemporary, multifaceted overview of the clinical and pathological presentations of urological conditions in people living with HIV (PLWHIV), based on articles published between 1989 and 2025. Conditions discussed include HIV-associated nephropathy (HIVAN), opportunistic genitourinary infections, malignancies such as Kaposi sarcoma and lymphoma, as well as non-infectious complications such as HIV-associated nephropathy and erectile dysfunction (ED). The review highlights the evolving epidemiology of these conditions in the cART era, with a noted decline in opportunistic infections but a rising burden of chronic kidney disease and malignancies, largely due to improved survival and ageing of the HIV-positive population. Pathological insights are explored and discussed, including mechanisms of HIV-associated renal injury, such as direct viral infection of renal epithelial cells and genetic predispositions linked to Apolipoprotein L1 (APOL1) variants. In addition, psychosocial factors, including anxiety, stress, stigma, and alcohol use, are discussed, as they may contribute to late presentation to clinical urology services. The review also considers the challenges faced in low and middle-income countries, the impact of HIV on urological services, and the important role of palliative care in advanced disease. Ultimately, this review underscores the need for early recognition, comprehensive diagnostic and surgical evaluation, and integrated social, psychological, and palliative management strategies tailored to the unique needs of PLWHIV. A deeper understanding of the interplay between HIV, cART, psychosocial determinants, and urological health is essential for improving patient outcomes and guiding future research in this evolving field.

## 1. Introduction

HIV is a retrovirus that attacks the body’s immune system, specifically the white blood cells called CD4 lymphocyte cells [1]. This pathophysiology means that PLWHIV are susceptible to opportunistic infections and malignancies due to this immune system dysregulation. Transmission of the virus is primarily via the invasive penetration of specific bodily fluids. If left untreated, HIV can progress to Acquired Immunodeficiency Syndrome (AIDS). According to the World Health Organisation (WHO), there are an estimated 39.9 million people living with HIV at the end of 2023, 65% of whom were originally or reside in Africa [2].

The most recent UK-wide estimate suggests that there are around 113,500 people living with HIV (PLWHIV), with the highest rates observed in London [3]. Globally, 53% of PLWHIV are female [4]. HIV rates are higher among certain groups worldwide, including young women in eastern and southern Africa, men who have sex with men (MSM), sex workers, injecting drug users (IDU), transgender individuals, and prisoners. However, rates of HIV among both heterosexual men and women are increasing, with approximately half of the people on HIV treatment in the UK having acquired the virus through heterosexual transmission, according to national data from 2023 [5]. Black Afro-Caribbean individuals accounted for 55.6% of people receiving a new diagnosis of HIV in 2023 [5]. Not only is the landscape of HIV acquisition changing, but the implications of an HIV diagnosis are also evolving. With the implementation of combination antiretroviral therapy (cART), HIV is no longer considered a death sentence as it was historically; instead, it is now managed as a chronic, treatable condition in outpatient settings, despite remaining incurable.

**Figure 1:**
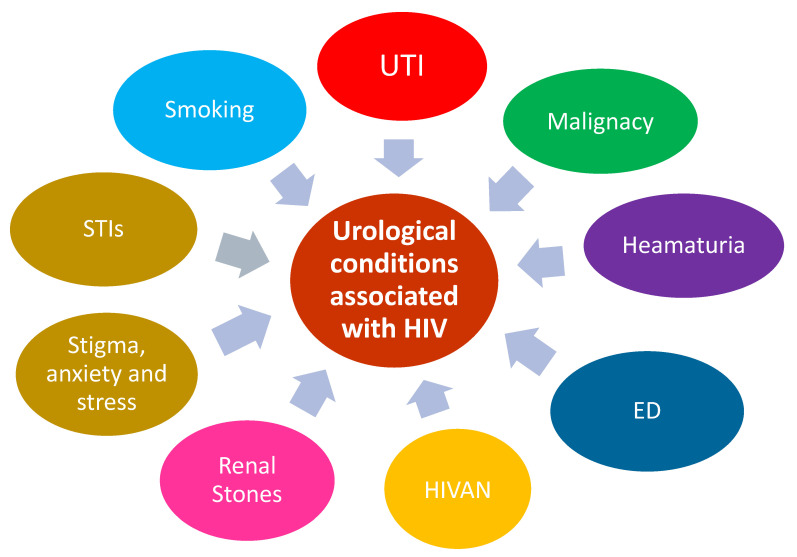
Shows urological conditions associated with HIV. UTI (Urinary Tract Infections), STI (Sexually Transmitted Diseases), HIVAN (HIV Associated Nephropathy), and ED (Erectile Dysfunction).

PLWHIV now commonly reach ages in their 70s and above, which in turn has led to changes in the surrounding landscape of diseases. With PLWHIV living longer, they potentially live to face the implications of their lifestyle choices, such as smoking and alcohol abuse. They may experience erectile dysfunction linked to poor cardiovascular health or bladder cancer linked to carcinogens in cigarette smoke. They are affected by the standard effects of ageing alongside the prolonged effects of being immunosuppressed or the long-term effects of being on cART. This article focuses on a wide range of urological conditions, ranging from urological infections such as urinary tract infections (UTI) to urological malignancies, and how PLWHIV are affected by such afflictions. The summary of urological conditions associated with HIV can be seen in Figure 1. Furthermore, it considers the nuanced differences in the presentation and treatment of such conditions compared with the general population. This article also addresses the stigma still surrounding PLWHIV, the potential psychological impact of a diagnosis and living with HIV and other potential Sexually Transmitted Infections (STIs), and the repercussions of a late presentation of advanced HIV disease.

While the epidemiology of people living with HIV (PLWHIV) is well-documented, there is a significant knowledge gap regarding the prevalence, incidence, and management of urological conditions within this population. As PLWHIV are living longer due to effective combination antiretroviral therapy (cART), new or previously under-recognised complications, including urological issues, may become more prevalent or clinically significant. This review is necessary to the following goals: (i) Identify and consolidate existing data on the frequency and types of urological conditions affecting PLWHIV. (ii) Reflect on the current literature in urological conditions, HIV, and social and medical management. (iii) Highlight gaps in knowledge and practice, thereby guiding future research priorities and improving patient care protocols for this population.

Importantly, the review also provides an excellent survey for updates on current research on HIV and urological conditions. This is important as at present, there is no universal consensus or standardised guidelines specifically tailored for screening and managing urological conditions in PLWHIV. Most recommendations rely on general population guidelines, with modifications based on clinical judgement considering the patient’s HIV status and immune function. In the cART era, the approach has shifted towards early detection and proactive management to prevent complications, but practices vary widely. Some experts advocate for targeted screening in high-risk groups (e.g., older PLWHIV, those with a history of smoking or recurrent infections), while others emphasise individualised care based on symptomatology and risk factors. More research and consensus-building efforts are needed to establish evidence-based protocols.

## 2. Methods

**Figure 2:**
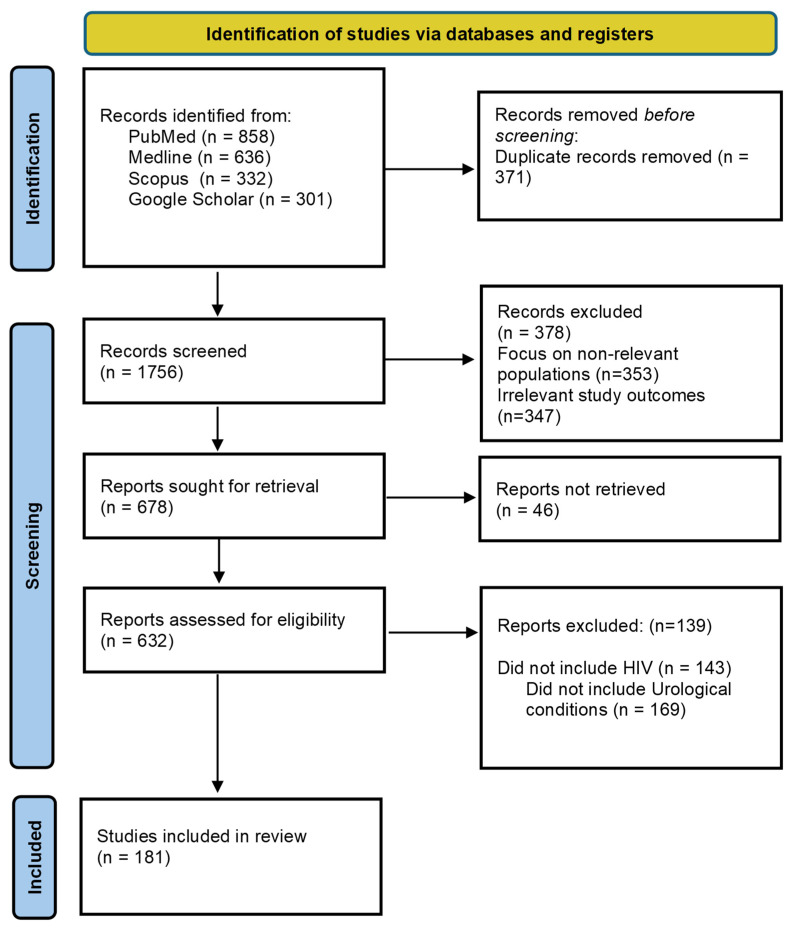
Diagram detailing the identification of studies via databases included in this narrative review.

A comprehensive literature search was conducted across PubMed, MEDLINE, Scopus, and Google Scholar to identify relevant studies published between 2000 and 2025. The search combined keywords, including but not limited to: “HIV,” “people living with HIV,” “urological infections,” “haematuria,” “urological malignancy,” “erectile dysfunction (ED),” “UTIs,” “prostatitis,” “epididymo-orchitis,” “Fournier’s gangrene,” “condyloma acuminatum,” “nephropathy,” “voiding dysfunction,” “lifestyle factors,” “smoking,” “alcohol,” “stigma,” “developing countries,” and “palliative care.” Reference lists of key articles and recent reviews were hand-searched to ensure comprehensiveness and to capture relevant grey literature (Figure 2).

### 2.1. Eligibility Criteria

Articles were included if they met the following criteria:

- Focused on urological manifestations or complications associated with HIV in adult populations (≥18 years).
- Published in US/UK English between 2000 and 2025.
- Presented original data, systematic reviews, narrative reviews, or meta-analyses that addressed epidemiological, clinical, pathological, psychosocial, management, or health services dimensions of urological conditions in PLWHIV.

Studies were excluded if they were case reports of singular clinical encounters without broader applicability, non-urological HIV-related topics, paediatric populations, or non-peer-reviewed commentaries or editorials.

### 2.2. Study Screening and Selection

Following de-duplication, records were screened in two successive stages. First, titles and abstracts were assessed for relevance according to the eligibility criteria. Articles deemed relevant were then retrieved in full text and assessed independently by two reviewers. Discrepancies in inclusion decisions were resolved through discussion or in consultation with a third senior reviewer where needed.

### 2.3. Synthesis Approach

Given the heterogeneity of study designs, populations, and outcomes across the corpus, a narrative synthesis was conducted. This approach allowed integration of quantitative prevalence estimates and qualitative psychosocial findings. Where possible, results were grouped by theme and clinical relevance and compared to general population benchmarks to delineate differences specific to PLWHIV. The synthesis aimed to identify patterns, gaps, and contextual nuances, especially pertinent to varied healthcare settings.

### 2.4. Quality Assessment

Although narrative reviews typically do not incorporate formal risk-of-bias scoring, methodological rigour was nonetheless appraised qualitatively. Preference was given to studies with clearly defined diagnostic criteria, transparent methods, and representative sampling.

## 3. HIV and Urological Manifestations

### 3.1. Haematuria

Haematuria is defined as blood in the urine and is further sub-classified as: microscopic haematuria (microhaematuria) and macroscopic haematuria (macrohaematuria or gross haematuria). Microhaematuria, also known as non-visible haematuria, is the presence of three or more red blood cells (RBCs) per high-power microscope field on a midstream, clean-catch urine sample [6], whereas macroscopic haematuria is that visible to the naked eye. Haematuria is a more significant concern in a population with HIV, owing to the following facts: (a) there is a higher prevalence in the HIV population when compared to the general population; (b) there is a wider range of differentials that must be considered in the HIV population. Recent studies suggest that the prevalence of haematuria in patients with HIV ranges from 18 to 50% [7,8], with greater haematuria as the CD4 count decreases [9], whereas the prevalence of haematuria is estimated to be 2.5–20% in the general population [10]. The main differentials causing haematuria in PLWHIV can be divided into these broad categories: pathology affecting the glomerulus (such as HIVAN, focal segmental glomerulonephritis and immune complex mediated glomerulonephritis) [11]; cART toxicity; opportunistic infections depending on CD4 count (such as Tuberculosis, Schistosomiasis and Cytomegalovirus) and urological malignancies. These differentials sit alongside the common causes of haematuria affecting all patients regardless of HIV status, including simple UTIs, urinary tract or kidney stones. Existing literature acknowledges that for the majority of PLWHIV who are young and asymptomatic with microscopic haematuria, a urological evaluation can be safely omitted in the presence of normal renal function and a benign urological history [12].

The diagnostic evaluation of haematuria in HIV-positive patients is similar to that of the general population, although a broader range of differential diagnoses must be considered. The workup for haematuria can include basic urinalysis and renal function blood tests, progressing to cystoscopy and renal biopsy depending on the clinical presentation. Patients may meet the two-week wait criteria for bladder cancer, which applies to those aged 45 and over with either unexplained visible haematuria without urinary tract infection, or visible haematuria that persists or recurs after successful treatment of a urinary tract infection [13]. Meeting these criteria would ultimately lead to a cystoscopy.

Due to the wide range of differentials which need to be considered, a comprehensive history and examination is crucial in order to observe or elicit clinical signs which may point to a particular diagnosis.

### 3.2. Anxiety

PLWHIV are more likely to experience elevated levels of anxiety compared to the general population [14]. A broad spectrum of anxiety disorders has been observed within this group, with adjustment disorder frequently occurring after an HIV diagnosis [14]. Anxiety in PLWHIV is often precipitated by critical moments in their clinical journey, including the initial diagnosis, the onset of illness due to opportunistic infections, a declining CD4^+^ T-cell count, or an increasing HIV-1 viral load. The Yale University AIDS Programme found that as many as 38% of people with HIV will encounter an anxiety disorder at some point in their lives [15]. Internalised stigma is a key determinant of anxiety surrounding HIV disclosure [16]. Worries about onward transmission of HIV are also a source of extensive anxiety for PLWHIV [17]. Anxiety surrounding PLWHIV and urological manifestations is treated the same as anxiety in the general population—either pharmacologically, non-pharmacologically, or a combination of the two. The first line of pharmacological treatment is selective serotonin reuptake inhibitors (SSRIs). However, for PLWHIV, interactions between SSRIs and cART must be considered carefully. Some SSRIs (namely sertraline and citalopram) have been shown to induce decreased metabolism, particularly in the setting of the ART ritonavir-based treatment regimens [18]. Moreover, some SSRIs exacerbate urological conditions pre-existing in large proportions of the HIV population, namely, erectile dysfunction. Non-pharmacological interventions (especially cognitive behavioural stress management interventions and cognitive behavioural therapy) are generally more effective than pharmacological interventions according to a systematic review conducted in 2011 [19].

### 3.3. Erectile Dysfunction (ED)

ED is defined as when one is unable to acquire an erection and/or unable to keep an erection long enough to have sexual intercourse [20]. The reported global prevalence of ED in the general population is highly variable because of population selection, erectile dysfunction criteria, and modalities to evaluate it [21]. The proportion of men experiencing ED is significantly higher in the HIV positive population (40–60%) [22,23], with this population typically being affected at a younger age when compared to the general population. When evaluating the aetiology of ED in men living with HIV, it is important to distinguish between causes common to the general population and those unique to this cohort. A fundamental consideration is whether the ED has an organic basis or is predominantly psychogenic in origin. As in other areas of clinical medicine, organic causes must be thoroughly investigated and excluded prior to attributing symptoms to psychological factors, although this is often difficult given that ED is frequently multifactorial in nature. Organic causes observed in both HIV-positive individuals and the general population include vasculogenic, neurogenic (central or peripheral), anatomical, and endocrine factors [24]. Among these, cardiovascular disease represents the most prevalent vasculogenic cause, while diabetes mellitus is the primary neurogenic contributor. Additionally, pharmacologic agents—such as antihypertensives, diuretics, and antidepressants—are well-documented contributors to ED, though the range of implicated medications is extensive [25].

A major contributing factor to ED in PLHIV is HIV viral replication itself, which can lead to increased inflammatory markers and endothelial dysfunction and mirrors mechanisms that parallel the pathophysiological processes observed in cardiovascular disease-related ED [26]. Furthermore, uncontrolled viral replication in the setting of delayed cART may lead to continued progression of endothelial damage, increasing the prevalence of ED [27]. Hence, the importance of early detection and diagnosis and rapid initiation of cART can reduce the likelihood of experiencing ED. However, whilst a delay in initiation of cART can lead to erectile dysfunction, some forms of individual cART therapies themselves can contribute to this ED, necessitating a balanced, holistic approach. A hospital-based, multicentric, cross-sectional study conducted in Tanzania showed that self-reported good adherence to cART was associated with ED in a multivariate logistic regression analysis [28]. Another study based in Spain elicited a statistically significant relationship between ED and protease inhibitor use [29]. cART contributing to ED and sexual dissatisfaction has also been demonstrated with integrase inhibitors, such as dolutegravir, in a Ugandan study [30].

In the general male population, testosterone plays a critical role in regulating male sexual function. It influences libido, erectile physiology, and overall sexual performance [31]. Different factors can contribute to ED, such as vascular, neurogenic, psychological, and endocrine factors (such as testosterone insufficiency) [31,32]. Importantly, testosterone deficiency (hypogonadism) is associated with decreased libido and, in some cases, ED; however, the relationship is complex. While testosterone therapy can improve sexual desire, it often does not fully restore erectile function when vascular or neurological pathology is present [32]. Among PLWHIV, testosterone insufficiency is significantly more prevalent. Studies suggest that 9–40% of HIV-infected men experience low testosterone, compared to 5–10% in the general population [33]. The mechanisms include hypothalamic–pituitary–gonadal axis dysfunction, chronic inflammation, opportunistic infections, and elevated sex hormone-binding globulin (SHBG) that reduce bioavailable testosterone [33]. Beltran et al. (2003) found that 16% of HIV-positive men had hypothyroidism, and 6.6% had subclinical forms, suggesting broader endocrine disruption [34]. Moreover, De Ryck et al. (2013) reported that 61.9% of HIV-positive men experienced ED, and 36.7% of those had testosterone deficiency [35]. Despite the fact that low testosterone contributes to reduced libido and erectile function in HIV, ED in this group is often multifactorial—linked to psychosocial stress, medication effects (antiretroviral therapy), and vascular dysfunction [36]. Consequently, screening for total and free testosterone is recommended for HIV-infected men presenting with sexual dysfunction [37]. Therefore, in the general population and PLWHIV, testosterone insufficiency plays a contributory, though not exclusive, role in ED. Testosterone replacement therapy (TRT) can improve libido and mood, but it should be integrated with management of comorbidities such as cardiovascular risk factors, medication side effects, and psychological stressors [38].

Psychological stress also plays an important role in erectile dysfunction in the HIV population. Pertinent psychological factors include fear of virus transmission, changes in body image and HIV distress and stigma [39]. Sexual issues, unfortunately, tend to be overlooked during consultations [40]. These dysfunctions, especially if undermanaged, further impair the quality of life and general health, interfering with intimate relationships and lowering the adherence to antiretroviral medications [41]. One study found that PLWHIV, on long-term treatment with cART, presented with alarming rates of depression/anxiety, which in turn is correlated with sexual and physical health problems [36]. It is difficult to say necessarily which comes first, the psychological distress or the sexual dysfunction, but they are undoubtedly closely intertwined and hence both need to be addressed sufficiently in order to treat any PLWHIV and current or potential partner holistically. The role of the multidisciplinary team is crucial in these cases in order to treat the patient comprehensively and improve patient outcomes.

Management of erectile dysfunction depends on the cause. It is crucial to take a detailed history, including an assessment of the mental impact of having HIV and ED on the patient. Organic causes such as cardiovascular disease, diabetes, low testosterone or medicinal side effects need to be excluded before settling on a psychogenic cause. It is often the case that the cause is multifactorial, and as such, management plans should reflect this by addressing all aspects of the biopsychosocial model. First-line pharmacological treatment for erectile dysfunction is a phosphodiesterase type 5 (PDE5) inhibitor such as sildenafil. Importantly, it must be checked as to whether there are any interactions with the patient’s cART regimen. PDE5 inhibitors interact with Ritonavir or Cobicistat; hence, some PDE5 inhibitors are contraindicated, whilst others need dose reductions [35]. The next treatment escalation after failing medical management would be injections into the penis to cause an erection. If all the above fail, then surgical implants or vacuum pumps are considered as a last resort. Alongside pharmacological treatments, lifestyle modifications promoting good cardiovascular health, such as smoking cessation and weight loss, are also important.

### 3.4. Malignancy

Access to cART to an increasing and ageing population of PLWHIV. According to the latest data from the Centres for Disease Control and Prevention (CDC), 54% of the nearly 1.1 million individuals diagnosed with HIV in the United States were aged 50 or older in 2022. Similarly, in the United Kingdom, almost half (49.8%) of those receiving HIV care in the same year were 50 and above. Although life expectancy for HIV-positive individuals has significantly improved, it is still shorter compared to that of HIV-negative people. There are ongoing discussions about whether HIV accelerates the ageing process. Elevated inflammatory processes in PLWHIV, even with low viral loads and stable CD4 counts, are linked to higher risks of cardiovascular, renal, neurocognitive, oncological, and osteoporotic conditions. As these health issues increase exponentially with age, they create a significant challenge to an ageing HIV population [42].

The incidence of AIDS-defining cancers (ADCs), such as Kaposi sarcoma, has declined with effective cART. However, non-AIDS-defining cancers (NADCs), including lung, liver, anal, and head and neck cancers, are now predominant among ageing PLWHIV [43]. This shift reflects both improved survival and the combined effects of ageing, chronic inflammation, and prolonged immune dysfunction. Low CD4 counts and incomplete immune recovery have consistently been associated with higher risks of both ADCs and infection-related NADCs [44]. In a multicentre cohort of PLWHIV with viral suppression for more than two years, higher time-updated CD4 counts were linked to significantly lower cancer incidence—adjusted incidence rate ratios declined from 1.0 at <350 cells/µL to 0.26 at ≥750 cells/µL [45]. Similarly, a recent study found that low baseline or nadir CD4 count and a CD4/CD8 ratio < 0.4 were strongly correlated with increased rates of infection-related cancers [46]. These findings suggest that while immune recovery reduces cancer risk, the relationship is most pronounced for malignancies with viral or infectious etiologies. Importantly, older PLWHIV often experience delayed HIV diagnosis and slower CD4 recovery, making them more prone to malignancy [47]. Thus, older PLWHIV with low CD4 counts represent a particularly high-risk group for cancer development. It is worth noting that as PLWHIV increasingly survive into older age, malignancy risk is driven not only by chronological ageing but also by cumulative immunodeficiency and incomplete immune restoration. Maintaining high CD4 counts (≥500–750 cells/µL) through early ART initiation, sustained viral suppression, and regular monitoring is key to mitigating cancer risk. Clinical programmes should incorporate malignancy screening, especially for HPV-, HBV-, and HCV-related cancers—targeting older individuals and those with persistently low CD4 counts. Immune status, therefore, remains a central and modifiable factor linking HIV infection, ageing, and cancer.

#### 3.4.1. Urological Malignancies in HIV Patients

It was well-established that there are specific cancers that can be found in HIV-positive individuals, which are known as AIDS-defining malignancies, including Kaposi’s sarcoma (KS), non-Hodgkin lymphoma, and invasive cervical cancer. Additionally, viral infections like human herpesvirus 8 (HHV8), Epstein–Barr virus (EBV), and human papillomavirus (HPV) can contribute to the development of AIDS-defining cancers [42]. Nowadays, after the introduction of cART, the incidence of these malignancies has significantly reduced [48]. However, chronic HIV infection is associated with a higher incidence of non-AIDS-defining cancers. Given the longer life expectancy of PLWHIV, it is important to assess cancer risks in this population, considering both age and duration of time since HIV diagnosis [42]. It was assumed that the increased cancer risk may be due to several factors, including immune system dysregulation caused by HIV, co-infections with cancer-causing viruses, and a higher prevalence of behaviours such as smoking, which increase cancer risk [42].

#### 3.4.2. Kaposi’s Sarcoma

KS is a multicentric tumour that often manifests as vascular skin or mucosal nodules. In some cases, it may remain confined to the skin, while in others it may affect the oral cavity, lymph nodes, or internal organs [49]. HHV8 is thought to play a central role in the development and progression of KS [49]. Diagnosis and management can be challenging, as KS may be mistaken for other skin conditions. Although KS of the urinary system is rare, HHV8 is often found in the urine of infected patients. In some cases, urethral lesions can cause urinary retention or obstruction [50]. A combination of systemic chemotherapy and cART is often required for advanced or metastatic KS. In advanced cases, which involve the urethral mucosa or penile skin, patients may require supra pubic catheterisation for symptom relief, and in rare instances, penectomy might be needed to remove invasive masses [51].

#### 3.4.3. Lymphoma

The incidence of lymphoma in HIV-positive patients is significantly higher than in the general population. This increased risk is likely due to several factors, including the transformative properties of HIV, immunosuppression and cytokine dysregulation caused by the infection, as well as opportunistic infections with lymphotropic herpesviruses like EBV and HHV8 [52]. These factors contribute to the development of lymphoma in PLWHIV at rates far exceeding those in HIV-negative individuals [52].

#### 3.4.4. Bladder Cancer

Although only a limited number of bladder cancer cases have been linked directly to HIV infection, multiple case reports document instances where HIV patients developed this type of cancer. In one study, 11 HIV patients with bladder cancer were observed, with a median age of 55. Six of these patients had identifiable risk factors for bladder cancer, and 10 of the 11 had Transitional Cell Carcinoma [53]. Treatments typically involved transurethral resection of the bladder tumour, followed by a combination of local and systemic therapies [53]. Another study involving 15 HIV patients with bladder cancer found that a high proportion of tumours (47%) were muscle-invasive, and 73% were of high histological grade [54]. Both studies showed that bladder cancer in PLWHIV tends to occur at a younger age and often presents with haematuria. The second study also noted more aggressive pathological features in these patients [54].

#### 3.4.5. Prostate Cancer

The true incidence of prostate cancer in people living with HIV is still unclear, as studies on the subject have yielded conflicting results. Some studies suggest that the rise in prostate cancer cases among PLWHIV may be linked to the global spread of PSA screening, mirroring trends in the general population [55]. However, other studies have indicated a lower incidence of prostate cancer in HIV-positive individuals, possibly due to HIV’s effect on inhibiting cancer cell growth or changes associated with cART [55]. Some researchers have also hypothesised that men with HIV receive PSA screening tests less frequently than the general population, which may explain the lower reported incidence [55]. Before cART became widely available, prostate cancer in HIV-positive patients was often associated with a severely compromised immune system, and androgen deprivation therapy (ADT) had poor outcomes, possibly due to a hypogonadal state [56]. Further research is needed to establish a clear link between HIV and prostate cancer, though treatment options for HIV-positive individuals should be consistent with those offered to the general population [56].

#### 3.4.6. Renal Cell Carcinoma (RCC)

The well-established risk factors for RCC include smoking, obesity, hypertension, and the von Hippel–Lindau (VHL) mutation, as well as occupational exposure and alcohol consumption [57]. Multiple studies showed that HIV-positive individuals are more likely than the general population to develop RCC, and they tend to be diagnosed at a younger age. However, many other aspects of RCC in HIV patients remain poorly understood [57]. Studies show that HIV-positive RCC patients have both shorter overall survival and worse progression-free survival compared to their HIV-negative counterparts, with a lower CD4+/CD8+ ratio being associated with poorer outcomes [58].

## 4. Alcohol and Smoking and Ultimate Impact on the Urological System

Rates of smoking and alcohol use are higher in PLWHIV when compared to the general population. Smoking rates are as high as 70% in PLWHIV in some studies [59], whilst the prevalence of alcohol abuse in PLWHIV can be as high 50% [60]. Smoking negatively impacts general and urological health. Smoking has the potential to shorten the life of a person taking HIV treatment by an average of six years and is far more harmful to the life expectancy of people living with HIV than well-managed HIV infection itself [61]. Evidence suggests that smoking-related illnesses, such as cardiovascular disease (cerebral vascular and myocardial infarctions), respiratory illnesses (such as COPD), and certain malignancies (such as lung and bladder cancer), contribute substantially to morbidity and mortality among HIV-infected patients [62]. Furthermore, smoking accelerates atherosclerosis, which in turn contributes to ED. Not only does smoking affect the cardiorespiratory systems and increase the risk of malignancy as it does in the general population, but it also affects the success of cART. Smokers with HIV on cART had poorer viral responses, poorer immunologic responses, greater risk of virologic rebound and more frequent immunologic failure in a large, longitudinal cohort study conducted in the USA [63]. Smoking status and, following that, cessation support, must be included in consultations with PLWHIV, as smoking is more detrimental to their health compared to the general population.

Moreover, alcohol abuse support and substance misuse support also need to be included in consultations with PLWHIV. Frequent alcohol users (two or more drinks daily) were 2.91 times more likely to present a decline of CD4 to ≤200 cells/μL, independent of baseline CD4^+^ cell count and HIV-1 viral load, antiretroviral use over time, time since HIV diagnosis, age and gender [64]. A cross-sectional analysis of HIV-1-infected drug users found that heavy alcohol users (defined as alcohol intake three to four or more times per week), who were receiving cART, were four times less likely to achieve an undetectable viral load and two times more likely to have CD4^+^ cell counts below 500 cells/μL than moderate drinkers or abstainers [65]. With CD4^+^ counts negatively impacted by alcohol, the risk of opportunistic infections is increased. With regards to urological infections, these can include more frequent episodes of prostatitis, epididymo-orchitis, UTIs and STIs. The risk of all malignancies, including urological malignancies, is also increased. Alcohol and substance misuse can increase risky behaviours, which may, in turn, also lead to an increase in urological infections and sexually transmitted infections.

## 5. Infections

Infections represent one of the common urological presentations among PLWHIV. Extensive detail for types of infection can be found in Table 1.

### 5.1. Urinary Tract Infections

Globally, urinary tract infections are an extremely common occurrence. A systematic review and meta-analysis, encompassing 38 studies and 981,221 individuals worldwide, found that the incidence of UTIs varied from 1.1 to 3.7% globally [66]. In the general population, it is women who are affected most frequently by urinary tract infections. In a population-based randomised household survey conducted in England in 2015, it was found that 37% of women reported at least one UTI in their lifetime [67]. UTIs are frequent in young sexually active women with reported incidence rates ranging from 0.5 to 0.7 per person-year [68], whereas in young men aged 18–24, the reported incidence is 0.01 per person-year [69]. The incidence decreases within the middle-aged population but then steadily rises in the over-65 population [70]. Over 10% of women older than 65 years reported a UTI within the past 12 months [71]. Age-related factors—malnutrition, poor bladder control, diabetes, among others—increase susceptibility both in the general and PLWHIV populations [72]. PLWHIV are also susceptible due to immunological decline [73], mucosal barrier dysfunction [74,75,76] and microbial virulence [72,77,78], in addition to the usual age-related physiological changes [79,80,81]. A study conducted in Northern Ethiopia found the frequency of UTI to be highest in the 35–44 years age group [82]. This frequency being higher in the middle-aged population may be reflective of the fact that, whilst HIV care has been transformed by antiretroviral therapies, resulting in more people living longer with the disease, there is a delay in the current data reflecting this, particularly in developing countries. Bacteriuria prevalence varies: 4–25.3% across locations, including Warsaw, India, and Nigeria [83,84,85]. In Tanzania, 12.3% of PLWHIV had bacteriuria, higher in females and those with CD4 < 200 cells/mm^3^ [72]. Multidrug resistance affected 45.9% of bacteriuric patients [72]. Bacteria that cause UTIs among HIV patients include *Escherichia coli*, *Enterococcus* species, *Pseudomonas aeruginosa*, *Proteus* species, *Klebsiella* species, and *Staphylococcus aureus* [86,87]. These are not dissimilar to those of the pathogens responsible for UTIs in the general population, which are most commonly: *Escherichia coli*, *Klebsiella pneumoniae*, *Proteus mirabilis*, *Enterococcus faecalis* and *Staphylococcus saprophyticus* [88]. See Table 1 for infection summary. Increase in susceptibility due to the following:

(1) Immunosenescence, the age-related decline of immune function, results from reduced T-cell renewal and increased terminally differentiated T cells [73]. In PLWHIV, chronic HIV stimulation or co-infections trigger persistent immune activation and inflammation, accelerating ageing [73]. CD4^+^ T cells are central to pathogen defence, including UTI pathogens [89]. PLWHIV with CD4^+^ < 200 cells/mm^3^ face increased UTI risk, often from multidrug-resistant organisms like Pseudomonas [89].
(2) HIV depletes mucosal CD4^+^ T cells, compromising barriers [74] and damaging gut mucosa, causing immune dysfunction and microbial translocation [74]. Chronic inflammation and loss of mucosal integrity foster bacterial colonisation [76].
(3) Urinary microbiome shifts in PLWHIV indicate increases in atypical and resistant organisms [72]. While *E. coli* remains common, *Pseudomonas aeruginosa* and *Enterococcus* spp. are more frequent [72]. Their virulence factors—adhesins, hemolysins, biofilms—enable persistent infections [77,78].
(4) Age-related urinary changes, including decreased bladder elasticity, urinary stasis, and comorbidities (catheters, diabetes, kidney disease), further increase UTI susceptibility [79,80,81]. Older adults may present atypically (confusion, delirium, incontinence, hypotension) [90]. Untreated UTIs risk cystitis, pyelonephritis, or sepsis [91]. PLWHIV presentations may be more severe due to immunosuppression [73]. Diagnosis requires urinalysis and cultures for atypical pathogens [91].

### 5.2. Prostatitis and Prostatic Abscess

Extensive detail for types of infection can be found in Table 1.

### 5.3. Sexually Transmitted Co-Infections

HIV and STIs commonly co-occur, particularly among individuals who are newly diagnosed with HIV [92]. Research has shown that people who test positive for HIV during STI screening have an average STI prevalence of 19.6%, highlighting the strong association between STIs and HIV transmission risk [93]. Moreover, new STIs are frequently acquired shortly after HIV infection. A study by Erbelding et al. found that, following a new HIV diagnosis, men typically contracted a new STI within 415 days and women within 176 days [94]. Importantly, STI co-infections are not limited to the early stages of HIV; they also occur throughout the course of infection. In fact, data from HIV outpatient clinics where most patients have lived with HIV for a significant period show an average STI prevalence of 14% [93]. A systematic review conducted in 2011 demonstrated that individuals receiving cART for HIV also exhibited a notable prevalence of a concurrent STI [93]. Across 14 studies that reported cART use, an average of 67.4% of participants were receiving treatment (standard deviation [SD] = 19.9; median = 71%) [93]. In studies where cART use was documented, the point prevalence of STIs was 16.2% (SD = 23.7), which was comparable to the 16.5% (SD = 13.3) observed in studies lacking cART data, a difference that was not statistically significant (t = 0.9) [93]. Additionally, among samples reporting cART use, there was no correlation between the proportion of participants on cART and the prevalence of STI co-infections (r = 0.013, not significant) [93].

Data from the late 1990s in the United States, when cART was being initiated earlier during HIV infection, also demonstrated relatively consistent rates of gonorrhoea among HIV-positive individuals, ranging from 7.6 to 14.3 cases per 1000 person-years [95]. A summary of STIs can be found in Table 2.

### 5.4. Syphilis (Treponema pallidum)

Syphilis, caused by the spirochete *Treponema pallidum*, is a significant co-infection in PLWHIV [96]. The interaction between HIV and syphilis is bidirectional: syphilitic ulcers can facilitate HIV transmission, and HIV can modify the clinical course of syphilis [87].

In PLWHIV, syphilis may progress more rapidly, and the immune response to *T. pallidum* can be impaired. Syphilis causes both increased concentrations of HIV in blood plasma and decreased CD4^+^ cells [96]. This can lead to atypical presentations and an increased risk of neurosyphilis. Moreover, syphilis, especially in its secondary, systemic stage, can heighten immune activation in host cells, influence the release of cytokines like TNF-α, and stimulate transcription factors such as NF-κB, thereby disrupting normal cell cycle regulation and promoting increased replication of HIV [96]. Neurosyphilis can occur at any stage of syphilis and may present with a variety of neurological symptoms.

#### Microbiological Spectrum

*T. pallidum* is a microaerophilic, Gram-negative spirochete that cannot be cultured in vitro, making diagnosis reliant on serological testing and direct detection methods [97]. In PLWHIV, serological responses may be blunted, necessitating careful interpretation of related syphilis serology test results. Syphilis manifests in multiple stages, with distinct clinical features in PLWHIV. Primary syphilis typically presents with a painless ulcer (chancre), though lesions may be multiple and atypical in immunocompromised individuals [98]. Secondary syphilis may involve a diffuse rash, mucous patches, condyloma lata, and systemic symptoms such as fever and lymphadenopathy [99]. PLWHIV may experience overlapping stages, more aggressive progression, and increased risk of neurosyphilis at any stage, often with cognitive changes, meningitis, or cranial nerve involvement [100].

Treatment remains essentially the same as in the general population, though PLWHIV may require closer follow-up. Serologic response should be monitored more closely in PLWH, as serofast states and treatment failures are more common [101].

### 5.5. Chlamydia trachomatis

Chlamydia infection is the most commonly reported STI globally, with over 127 million cases annually [102]. PLWHIV, particularly young sexually active individuals and MSM, show higher prevalence due to overlapping risk factors [102]. *Chlamydia trachomatis* is an obligate intracellular Gram-negative bacterium [103]. It evades immune clearance and may establish chronic infections, contributing to persistent inflammation and increased HIV viral shedding [103]. In men, Chlamydia may cause urethritis, proctitis, or epididymo-orchitis, often with dysuria and discharge. In women, it is commonly asymptomatic but can lead to cervicitis or pelvic inflammatory disease [104]. Rectal infections, common in MSM, may be asymptomatic or, in some cases, can mimic the symptoms and signs of an inflammatory bowel disease [105].

### 5.6. Neisseria gonorrhoeae

Gonorrhoea is also a commonly reported STI globally, with over 82.4 million new infections globally in 2020 [106]. Gonorrhoea is a major co-infection in PLWH [95]. Infection increases both susceptibility to and transmission of HIV, with genital, rectal, and pharyngeal sites frequently affected [95]. *N. gonorrhoeae* is a Gram-negative diplococcus with pili and outer membrane proteins that promote epithelial adherence and invasion. It induces local inflammation, which enhances mucosal HIV shedding [107]. Gonorrhoea may present with urethritis, dysuria, purulent discharge, or proctitis. Many rectal and pharyngeal infections are asymptomatic. In women, it can cause PID and infertility [108].

### 5.7. Mycoplasma genitalium

*M. genitalium* is increasingly recognised as a cause of non-gonococcal urethritis and cervicitis. Prevalence is higher in PLWH, especially those with urethral symptoms [109]. This organism lacks a cell wall, rendering β-lactam antibiotics ineffective. It adheres to epithelial cells and induces chronic inflammation, which may prolong viral shedding in HIV-positive patients [110]. Symptoms include urethritis, dysuria, and discharge in men and cervicitis, pelvic pain, and abnormal bleeding in women. Persistent infection is common and often resistant to standard therapies [111].

### 5.8. Human Papillomavirus (HPV)

HPV is highly prevalent in PLWH. MSM and women with HIV have higher rates of anal and cervical HPV, respectively [112]. Immunosuppression leads to more persistent infections and a higher risk of malignancy [112]. HPV is a non-enveloped DNA virus that infects epithelial cells. High-risk types [16,18] are associated with malignancy, while low-risk types [6,11] cause condylomas [113]. HIV impairs clearance and promotes viral persistence [113]. HPV infection may be asymptomatic or present with genital or anal warts. High-risk types are associated with intraepithelial neoplasia and cancers of the cervix, anus, and penis, especially in PLWH with low CD4^+^ counts [114].

### 5.9. Epididymo-Orchitis

Epididymo-orchitis is a notable urological complication among PLWH. While comprehensive epidemiological data are limited, case reports have highlighted its occurrence in this population. The immunosuppressed state in PLWH predisposes them to infections by atypical and opportunistic pathogens. While common causative agents include *Neisseria gonorrhoeae* and *Chlamydia trachomatis* [115], PLWH are also susceptible to infections by organisms such as *Plesiomonas shigelloides* [116], as reported in a 2001 case study, and *Haemophilus influenzae,* as reported in a 1994 report [117]. PLWH may present with typical symptoms of epididymo-orchitis, including acute onset unilateral scrotal pain, erythema and swelling [118]. Patients may complain of urethral discharge or urethritis as well as symptoms in keeping with a urinary tract infection, such as dysuria, urgency and frequency [119]. Alternatively, symptoms of urethritis, urethral discharge and a urinary tract infection may be entirely absent [115]. However, due to immunosuppression, they may also experience atypical presentations or rapid progression to complications such as abscess formation or systemic infection. The aforementioned case reports highlight the necessity for clinicians to maintain a high index of suspicion and consider a broad differential diagnosis in this population [116,117]. Epididymo-orchitis is a clinical diagnosis that is primarily made based on presenting history, risk of sexually transmitted infections (STIs), physical examination findings and preliminary investigations such as urethral swab for culture and nucleic acid amplification test (NAAT), mid-stream urine specimen for microscopy and culture, screening for other STIs including blood-borne viruses and blood tests such as full blood count, white cell count and C-reactive protein [115]. For patients who may present with suspected tuberculous epididymo-orchitis, three early morning urine samples for acid and alcohol-fast bacilli are required [120]. Additionally, a chest X-ray, intravenous urography, renal tract ultrasound scan and a biopsy may be indicated [120].

### 5.10. Fournier’s Gangrene

Fournier’s gangrene (FG) is rare but more prevalent in PLWHIV, with increased mortality due to delayed presentation and comorbidities like diabetes [121]. FG is a polymicrobial necrotising fasciitis often caused by aerobic and anaerobic organisms, including *E. coli*, *Streptococcus*, and *Bacteroides*. HIV-related immunosuppression exacerbates infection severity [122]. Patients present with perineal pain, swelling, erythema, crepitus, and systemic signs of sepsis. Rapid progression necessitates urgent recognition [123].

### 5.11. Condyloma Acuminatum

These genital warts, caused by HPV types 6 and 11, are more frequent and extensive in PLWH due to impaired immune surveillance [124]. HPV infects basal epithelial cells, causing exophytic lesions. In PLWH, immune dysfunction permits rapid lesion growth and recurrence [125]. Lesions appear as soft, cauliflower-like growths on the external genitalia, perianal area, or mucosal surfaces. They may cause discomfort, bleeding, or psychosocial distress [126].

### 5.12. Fungal Infections

Fungal urinary infections are common in PLWH with CD4^+^ < 200 [127]. *Candida* spp. are most frequent, but systemic fungi like *Cryptococcus* or *Histoplasma* may involve the urogenital tract [127,128]. Candida colonises mucosa and invades tissue when immune defences are compromised. Systemic fungi may reach the urinary tract hematogenously [129]. Candiduria is usually asymptomatic but can cause cystitis or pyelonephritis. Cryptococcal or histoplasmic prostatitis may mimic bacterial prostatitis with fever, perineal pain, and urinary symptoms [130].

**Table 1:** Summary of urinary tract infections.

Infection	Diagnostic Consideration	Clinical Presentation	References
UTI	Can occur in up to 25%. Drug resistance may reach 45%. Immunosuppression increases the risk of UTI.	Can be atypical presentations. In the elderly, it may lead to confusion. Can present as sepsis.	[72,73,74,75,76,77,78,79,80,81,82,83,84,85]
Prostatitis and prostatic abscess	These are significant urological complications in PLWHIV. Intra-prostatic reflux and ascending urethral infections lead to the majority of cases. Common risk factors: surgery, biopsy, cystoscopy and catheterisation, Genito-urinary infections, high-risk sexual behaviour and benign prostatic hypertrophy. Common pathogens are *Escherichia coli*. Others include *Enterobacter*, *Klebsiella*, *Proteus* species, *Pseudomonas aeruginosa* and STIs, such as *Chlamydia trachomatis* and *Neisseria gonorrhoeae*.	Can be atypical, such as perineal discomfort, urinary frequency, and dysuria, these infections may progress rapidly to systemic infection. Therefore, a high index of suspicion is necessary. Accurate diagnosis requires a combination of clinical assessment and the use of transrectal ultrasound and CT scans. Microbiological analysis, including urine and blood cultures, is essential for identifying the causative organisms and guiding antibiotic therapy.	[131,132,133,134,135]
Epididymo-orchitis	Common pathogens: *Neisseria gonorrhoeae* and *Chlamydia trachomatis*. PLWHIV are also susceptible to infections by organisms such as *Plesiomonas shigelloides* and *Haemophilus influenzae*. Clinical diagnosis combined with investigations such as urethral swabs. Tuberculosis can be a cause, and prompt investigations will be needed.	Can present with acute onset unilateral scrotal pain, erythema and swelling, urethral discharge or urethritis in keeping with a urinary tract infection, such as dysuria, urgency and frequency (can be asymptomatic). Therefore, it can lead to abscess or sepsis- a high index of suspicion is needed and consider other differential diagnosis in PLWHIV.	[115,116,117,118,119,120]
Condyloma Acuminatum	Genital warts, caused by HPV types 6 and 11.	Lesions appear as soft, cauliflower-like growths on the external genitalia, perianal area, or mucosal surfaces. They may cause discomfort, bleeding, or psychosocial distress.	[124,125,126]
Fungal Infections	*Candida* spp. is most frequent. Systematic *Cryptococcus* or *Histoplasma* may involve the urogenital tract.	Cryptococcal or Histoplasmic prostatitis may mimic bacterial prostatitis with fever, perineal pain, and urinary symptoms.	[127,128,129,130]

**Table 2:** Summary of sexually transmitted infections.

Infection	Diagnostic Consideration	Clinical Presentation	References
Syphilis	Syphilitic ulcers can facilitate HIV transmission, and HIV can modify the clinical course of syphilis. It also increased concentrations of HIV in blood plasma and decreased CD4^+^ cells. This can lead to atypical presentations and an increased risk of neurosyphilis. The secondary systemic stage can heighten immune activation in host cells, promoting increased replication of HIV. Neurosyphilis can occur at any stage of syphilis.	Primary syphilis presents with a painless ulcer (chancre), though lesions may be multiple. Secondary syphilis may involve a diffuse rash, mucous patches, condyloma lata, and systemic symptoms such as fever and lymphadenopathy. There may be overlapping stages and increased risks of neurosyphilis at any stage.	[96,97,98,99,100,101]
*Chlamydia trachomatis*	Chlamydia is the most commonly reported STI globally, with over 127 million cases annually. PLWH, particularly young sexually active individuals and MSM, show higher prevalence due to overlapping risk factors.	Leads to chronic infections. In men, it may cause urethritis, proctitis, or epididymo-orchitis, with dysuria and discharge. In women, it is commonly asymptomatic but can lead to cervicitis or pelvic inflammatory disease. Rectal infections, common in MSM, may be asymptomatic or mimic inflammatory bowel disease.	[102,103,104,105]
*Neisseria gonorrhoeae*	Gonorrhoea is a major co-infection in PLWH. Infection increases both susceptibility to and transmission of HIV, with genital, rectal, and pharyngeal sites frequently affected.	Present with urethritis, dysuria, purulent discharge, or proctitis. Many rectal and pharyngeal infections are asymptomatic. In women, it can cause pelvic inflammatory diseases and infertility.	[106,107,108]
*Mycoplasma genitalium*	Recognised as a cause of non-gonococcal urethritis and cervicitis. Prevalence is higher in PLWHIV, especially those with urethral symptoms. It adheres to epithelial cells and induces chronic inflammation, which may prolong viral shedding.	Symptoms include urethritis, dysuria, and discharge in men and cervicitis, pelvic pain, and abnormal bleeding in women. Persistent infection is common and often resistant to standard therapies.	[109,110,111]
Human papillomavirus (HPV)	HPV is highly prevalent in PLWHIV. MSM and women with HIV have higher rates of anal and cervical HPV, respectively. Immunosuppression leads to more persistent infections and a higher risk of malignancy.	HPV infection may be asymptomatic or present with genital or anal warts. High-risk types are associated with intraepithelial neoplasia and cancers of the cervix, anus, and penis, especially in PLWHIV with low CD4^+^ counts.	[112,113,114]

## 6. HIV Associated Nephropathy (HIVAN)

HIVAN often presents with heavy proteinuria and a decline in kidney function that can progress to end-stage renal disease (ESRD). Clinically, HIVAN typically unfolds with rapidly worsening renal function and significant protein loss in the urine—usually more than 3–4 g per day [136,137]. The underlying pathology is collapsing focal segmental glomerulosclerosis (FSGS). In this condition, parts of the kidney’s filtering units (glomeruli) collapse, podocyte cells abnormally proliferate, and the tubules develop microcysts alongside inflammation in the supporting tissue [138]. HIV-1 directly contributes by infiltrating kidney epithelial cells in the tubules and glomeruli, even though these cells lack the classical viral entry receptors. Instead, the virus may exploit indirect routes via immune cells or alternative receptors such as DC-SIGN or DEC-205 [139]. A definitive diagnosis requires a kidney biopsy, which reveals the characteristic collapsing glomeruli and tubular changes [140].

Genetics also play a key role. In individuals of African ancestry, the risk is markedly higher due to variants in the APOL1 gene. People carrying two high-risk alleles (G1 or G2) are up to 29 times more likely to develop HIVAN compared with others [140,141]. Additional risk factors include advanced HIV infection (low CD4 counts, high viral load), male sex, and comorbidities such as hypertension or diabetes. These conditions have become more relevant as PLWHIV live longer on cART [142]. Importantly, thanks to the effectiveness of modern cART, HIVAN has become much less common. Still, the overall burden of chronic kidney disease (CKD) in PLWHIV remains high, largely due to ageing, other medical conditions, and the long-term effects of cART itself [143]. This makes vigilant screening and timely intervention essential.

Importantly, cART can cause urinary tract disorders and renal damage, mainly due to nephrotoxic effects of nucleotide and nucleoside reverse transcriptase inhibitors (NRTIs). Long-term use of tenofovir disoproxil fumarate (TDF) is linked to acute tubular necrosis, Fanconi syndrome, interstitial nephritis, and proteinuria, likely due to mitochondrial dysfunction in proximal renal tubule cells. TDF may also cause chronic kidney disease (CKD) and proteinuria [144,145,146,147,148,149,150,151,152,153,154,155,156,157]. Although TDF toxicity can improve after stopping the drug, some renal decline may persist [148]. Other NRTIs like stavudine and lamivudine, and protease inhibitors such as ritonavir and lopinavir, have also been linked to renal impairment [148,149]. Indinavir is associated with crystalluria and acute renal failure [149]. Regular monitoring of renal function is essential, and switching to tenofovir alafenamide (TAF) is recommended for high-risk patients due to its better renal safety [150].

Treatment focuses first on cART, which not only suppresses HIV replication but can also prevent or even reverse kidney injury [151]. ACE inhibitors or ARBs are often added to reduce proteinuria and slow disease progression [152]. In some cases, when kidney function continues to decline despite ART and ACE/ARB therapy, a short course of corticosteroids may help stabilise renal function. However, the supporting evidence is limited, and their use must be cautious [153].

## 7. Kidney Stones

PLWHIV are at increased risk of developing kidney stones (urolithiasis). In individuals without HIV, the main risk factors are usually linked to diet (such as excess calcium, oxalate, or uric acid intake), dehydration, and certain metabolic or endocrine disorders, including hyperparathyroidism, gout, and obesity [154,155]. In contrast, for PLWHIV, the picture is more complex. cART regimens, particularly those containing protease inhibitors (PIs), have greatly improved control of HIV suppression and transformed outcomes, but these drugs have also been associated with kidney stone formation.

Indinavir, one of the earliest PIs, was strongly linked to urolithiasis, with up to 20% of patients developing stones during treatment. These stones are unusual because they can crystallise directly from the drug itself, forming radiolucent stones that do not appear on plain X-rays. This often leads to delays in diagnosis and appropriate management [156]. Although the use of indinavir has declined, other PIs remain in clinical practice, and some still carry a significant risk of nephrolithiasis, namely, Atazanavir. Large cohort studies have shown that patients on Atazanavir, particularly when boosted with Ritonavir, have more than a tenfold increased risk of kidney stones compared with patients on other PIs [157]. Stones composed entirely of Atazanavir have been retrieved and analysed, confirming that the drug itself can crystallise and aggregate in the urinary tract [158].

Interestingly, case reports also describe kidney stones made up entirely of Ritonavir, sometimes occurring years after the drug had already been discontinued [159]. This highlights the potential for long-lasting metabolic effects of cART and underlines the need for ongoing vigilance. Unsurprisingly, the overall risk is further increased in PLWHIV who also have traditional stone risk factors such as obesity, gout, or hyperparathyroidism [154,155].

Clinically, kidney stones in PLWHIV present much like in the general population, with flank pain, haematuria, or recurrent urinary tract infections. However, drug-induced stones present diagnostic challenges. Because many are radiolucent, they may not be detected on standard X-rays, making ultrasound or CT imaging essential for accurate diagnosis [160]. Recurrent stone formation is also more common, particularly if the causative cART drug remains part of the treatment regimen.

Management requires a holistic and collaborative approach. Acute episodes often need standard measures such as analgesia, hydration, and in some cases, surgical procedures like ureteroscopy or lithotripsy. Prevention, however, is just as important. This may involve reviewing the cART regimen with infectious disease specialists, and in selected cases, switching from Atazanavir or Indinavir to alternative drugs to lower recurrence risk and always balancing this against the need to maintain viral suppression. Supportive strategies such as maintaining good hydration, modifying diet to reduce stone risk, and monitoring renal function should also form part of long-term care.

## 8. Psychological Stress and Stigma

Addressing all limbs of stigma (social, institutional and internalised) will act to reduce anxiety, particularly surrounding HIV disclosure. Therefore, in consultations with PLWHIV, it is necessary to address their psychological needs and screen for common disorders like anxiety and depression in order to intervene with both pharmacological and psychological support before any potential anxiety and depression worsen. Fundamentally, HIV care provides a holistic approach to a diagnosis of HIV, and the repercussions of the diagnosis affect every aspect of the person’s life, whether that be their self-image, their relationships, or how they are viewed by society. Whilst some may argue that this is a problem of the past, recent surveys evidence that these issues are still very much relevant. In 2022, a survey conducted in the UK by Positive Voices found that one in three PLHIV reported low self-esteem; one in ten have not shared their HIV status with anyone aside from healthcare staff; and almost half of the survey respondent reported feeling ashamed about their diagnosis [161].

## 9. HIV and Low- and Middle-Income Countries (LMICs) and Overall Impact in Urological Services

The global burden of HIV disproportionately affects the developing world and LMICs, with the highest incidence in sub-Saharan Africa, Southeast Asia, and parts of Latin America. Nearly 67% of all PLWHIV reside in sub-Saharan Africa according to data from 2021 [162]. A study conducted in sub-Saharan Africa found high rates of transmission to cohabiting sexual partners due to late diagnosis [163]. Late diagnosis of HIV increases the risk of morbidity, mortality, and HIV transmission. Due to limited healthcare infrastructure, late diagnosis and progression to AIDS are more common in the developing world.

In sub-Saharan Africa, particularly, 1.5 million PLWHIV live more than one hour’s drive from the nearest health facility offering treatment [164]. As a consequence, there is an increased risk of HIV-associated urological complications. Common urological manifestations amongst PLWHIV include a higher prevalence of UTIs due to poor sanitation and immune suppression, as well as genitourinary tuberculosis, which remains endemic in many HIV-affected regions [83,165]. Urological malignancies such as KS, bladder cancer and penile cancer are also more commonly seen in advanced HIV infection, partly due to co-infections like HPV and schistosomiasis [166,167]. Unfortunately, similarly to the UK but to a further extent relating to cultural reasons, stigma related to both HIV and urological symptoms discourages patients from seeking timely medical attention, further delaying diagnosis and treatment [168]. Defined as ‘a strong feeling of disapproval that most people in a society have about something’ [169], there are three types of stigma in PLWHIV: social stigma is negative stereotyping of PLWHIV; institutional stigma occurs in healthcare via judgmental practices; internalised stigma causes shame, guilt, and secrecy, leading to silence and denial [170]. Stigma delays healthcare engagement, increasing transmission and advanced disease; in Zimbabwe, most avoid HIV testing due to stigma-driven reluctance [170]. Late presentations in respect of HIV and urological issues, especially, are linked to poor outcomes and increased morbidity and mortality [171]. With both overlapping conditions having their own stigma, this cumulative stigma often means that PLWHIV are even more reluctant to present for urological consultation or may underreport particular issues to healthcare professionals (such as erectile dysfunction or abnormal discharge from genitals) [172]. Stigma needs to be recognised and addressed. There is a great importance and necessity to educate healthcare professionals to empower PLWHIV, support them psychologically and change community perceptions about PLWHIV [173]. By educating healthcare professionals, it can be ensured that all aspects of PLWHIV healthcare and welfare are addressed from a biological, psychological and social point of view. Public health campaigns also provide a useful platform to filter information and dispel misconceptions about HIV and PLWHIV to the general population. There is also a role for peer-led support groups in reducing stigma and ensuring PLWHIV have a reliable support network. A systematic review and meta-analysis of peer-support groups found that there was a significant retention in care, better antiretroviral adherence and superior viral suppression when PLWHIV had access to peer-led support compared to not having any [174].

Importantly, in LMICs and developing countries, the intersection of HIV, immune suppression (low CD4), ageing PLWHIV, and urological conditions (renal disease, stones, obstructive uropathy, urological cancers) poses a growing challenge that may exceed the capabilities of most health authorities in such countries. The pressing issues include late diagnosis of HIV, low CD4 nadir, limited urology workforce and infrastructure, inadequate integration of HIV and urological/renal care, and lack of data. Proposed resolutions focus on early ART/immune recovery, strengthening urology and nephrology capacity, integrating care pathways, pragmatic screening, building local research and addressing financing/access barriers. Because immune status (CD4 counts) modulates risk of malignancy and renal/urological disease, the same mechanisms discussed for malignancy (age + immunodeficiency) apply in urology as well. Investment in research will be an essential step to fully understand HIV-associated urological conditions in low- and middle-income countries (LMICs). Most available data come from high-income settings, leaving major gaps in understanding the epidemiology, clinical patterns, and outcomes in resource-limited contexts. Local research is essential to identify population-specific risk factors, such as delayed HIV diagnosis, low CD4 recovery, and co-infections and to evaluate feasible screening and treatment models. Strengthening research capacity in LMICs will guide evidence-based policies, improve integration of HIV and urological care, and ensure equitable access to diagnostics and interventions tailored to local healthcare realities and resource constraints.

## 10. Palliative and Social Care in Patients with HIV and Fatal Urological Conditions

According to the WHO, palliative care is an approach that improves the quality of life of patients who are facing problems associated with life-threatening illness. It prevents and relieves suffering through the early identification, correct assessment and treatment of pain and other problems, whether physical, psychosocial or spiritual [175]. HIV has transformed from being an inpatient-based terminal fatal condition to an outpatient-based chronic treatable condition. Whilst there has been a shift in life expectancy in PLWHIV due to the implementation of antiretroviral therapy, the life expectancy of PLWHIV is varied and multifactorial. It is influenced by many factors, including CD4^+^ count at diagnosis and age at diagnosis. A recent study conducted across North America and Europe found that life expectancy for PLWHIV who have been on cART is only somewhat lower than that in the general population [176]. It must be acknowledged, though, that there are very few studies that look at quality of life, with the literature predominantly focusing on the numerical increase in life expectancy. Moreover, whilst the overall population of PLWHIV are living longer in the developed world, PLWHIV who have low CD4^+^ at diagnosis and delayed initiation of antiretroviral therapy do not have such positive outcomes. There is still a role for social and palliative care in this population. Renal failure is a significant cause of morbidity and mortality in PLWHIV. Opportunistic infections and urological malignancies can also be fatal, particularly in those who are not on cART or who have not been compliant. A recent study found that there was an unmet need for palliative care for PLWHIV and that it is a crucial element that needs to be integrated in order to prevent health-related suffering [177]. The importance of advanced care planning is evident in many other chronic conditions that may result in deterioration. With suitable early palliative care input, patients and their families can be supported both physically and psychologically, and unnecessary suffering can be prevented. The British HIV Association (BHIVA) have incorporated palliative care within the ‘Standards of Care for People Living with HIV (2018)’, which can be easily implemented across all healthcare settings [178]. Similarly, social care and support are crucial for the holistic care of PLWHIV.

## 11. How to Improve Urology Service for PLWHIV

It is clear from the above discussion that urology services for PLWHIV require contributions from a broad range of healthcare professionals. An integrated multidisciplinary model is essential, involving collaboration between urologists, infectious disease specialists, nephrologists, oncologists, psychologists, and palliative care teams.

Combined clinics embracing a multidisciplinary care model, such as HIV Urology joint clinics, can reduce fragmentation of care and minimise stigma, while clear referral pathways from HIV clinics to urology services are necessary to enable the early detection and management of renal and genitourinary conditions.

Screening and early detection represent another important pillar of care. All PLWHIV should undergo routine urine dipstick testing and renal function monitoring to identify proteinuria, haematuria, or early renal dysfunction, while access to ultrasound and other imaging modalities is crucial for the timely recognition of obstructive uropathy, urolithiasis, or malignancy. Prostate, bladder, and testicular examinations should also be incorporated into long-term follow-up, given the elevated cancer risks in this population. In well-resourced centres, APOL1 genotyping may be considered in high-risk populations of African ancestry to stratify susceptibility to HIV-associated nephropathy.

Adequate resources must also be directed towards developing robust clinical and surgical services. This includes access to minimally invasive surgery such as endourology, laparoscopy, and laser stone management, supported by strict infection control measures. Preoperative planning should involve HIV physicians to address potential antiretroviral therapy interactions, bleeding risks, and immunosuppression. In tertiary and well-equipped centres, dedicated pathways for HIV-related malignancies such as Kaposi sarcoma, lymphoma, penile cancer, and anal cancer should be established, while renal replacement therapies, including dialysis and transplantation, should be made available without discrimination, with transplant programmes integrating HIV-specific expertise.

Psychosocial support and stigma reduction are equally critical elements of a high-quality urology service for PLWHIV. Counselling services addressing anxiety, stigma, sexual health, fertility, and body image should be included, alongside support for alcohol and substance use, which may otherwise delay presentation and complicate management [179]. Training staff in confidentiality and fostering non-judgmental environments are crucial to encourage timely engagement with services. In resource-limited countries, where the prevalence of HIV remains high, innovations are needed to enhance access to urological care. Examples include the use of point-of-care diagnostics such as dipstick testing and portable ultrasound to facilitate early detection, as well as training general practitioners and HIV clinicians in basic urological assessment where specialist urologists are scarce. Strengthening supply chains for essential drugs such as cART and ACE inhibitors, together with urological equipment, is vital. Integration of palliative care services is also essential for patients with advanced urological malignancies or end-stage renal disease in settings where curative options are limited.

**Figure 3:**
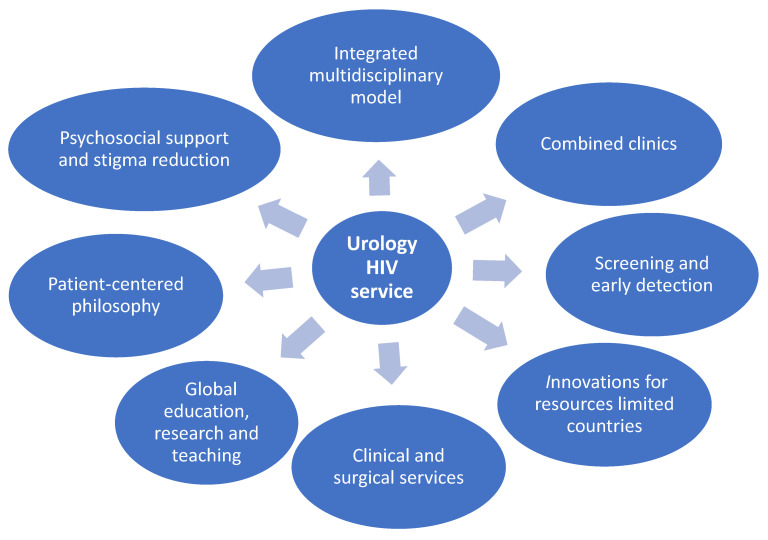
This figure represents an attempt to improve the urology-HIV service. Urology services for PLWHIV should adopt an integrated multidisciplinary model with combined clinics and clear referral pathways. Priorities include routine screening, imaging, and cancer surveillance, supported by advanced surgical care and equitable access to dialysis and transplantation. Psychosocial support, stigma reduction, and confidentiality training are essential. In resource-limited settings, innovations such as point-of-care diagnostics and workforce training are crucial. Globally, education, research, and patient-centred approaches ensure safe, equitable, and stigma-free care.

From a global health perspective, there is an urgent need for sustained efforts in education, research, and training. Continuous education of urologists should focus on evolving HIV therapies and their drug–drug interactions. Research networks should be developed to investigate urological complications of HIV in diverse populations, while HIV Urology modules should be incorporated into training curricula for urologists, particularly in regions with high HIV prevalence. Ultimately, urology services for PLWHIV must evolve towards a patient-centred philosophy, moving beyond a purely disease-based model to a holistic approach in which HIV is managed as a chronic condition alongside urological disease (Figure 3). Patients should feel safe, respected, and empowered to seek care early, with access to advanced therapies that are equitable and stigma-free, comparable to those available to HIV-negative individuals. A practical example of such an approach is demonstrated by the HIV metabolic clinic established at Milton Keynes University Hospital, which has successfully integrated patient-centred principles for over a decade and highlights the feasibility of this model in routine practice [180,181].

## 12. Conclusions

This narrative review synthesises the urological manifestations, complications, and management considerations in PLWHIV. Despite HIV being a chronic, manageable condition, it continues to impact urological health, influenced by immune status, comorbidities, cART, lifestyle, and healthcare access. Late presentation, often driven by stigma, leads to delayed diagnosis, worse outcomes, and psychological burden. Anxiety and internalised stigma further contribute to conditions such as erectile dysfunction, highlighting the need for integrated psychological support. HIV is associated with haematuria, infections (UTIs, prostatitis, STIs), malignancies (KS, bladder, prostate cancer), and non-infective complications (HIVAN, voiding dysfunction), often worsened by immunosuppression and cART toxicity. Integrated, patient-centred care—including prevention, early diagnosis, multidisciplinary management, lifestyle modification, and palliative support—is essential, alongside research on longitudinal outcomes and service integration. Importantly, in LMICs, these challenges are magnified by systemic barriers, including limited access to diagnostic facilities, specialist urological care, and essential medications. In such countries, late presentation with advanced immunosuppression, increasing susceptibility to urological infections, renal complications, and malignancies. Co-infections such as tuberculosis, schistosomiasis, and viral hepatitis remain major contributors to urological morbidity. Inadequate infrastructure, shortages of trained urologists, and fragmented health systems further compound these issues. Addressing urological health in LMICs, therefore, requires a comprehensive approach: strengthening primary healthcare systems, integrating urology and HIV services, promoting early HIV diagnosis and cART initiation, and expanding access to low-cost diagnostic tools. Culturally sensitive education and stigma reduction programmes are equally critical to improve care-seeking behaviours and treatment adherence. Investment in local research is also essential to better understand the burden and characteristics of HIV-related urological disease in LMICs and to develop context-specific management strategies.

## Data Availability

This narrative review and all data are included in this review.
